# RASopathic Skin Eruptions during Vemurafenib Therapy

**DOI:** 10.1371/journal.pone.0058721

**Published:** 2013-03-14

**Authors:** Jeannine D. Rinderknecht, Simone M. Goldinger, Sima Rozati, Jivko Kamarashev, Katrin Kerl, Lars E. French, Reinhard Dummer, Benedetta Belloni

**Affiliations:** Department of Dermatology, University Hospital of Zurich, Zurich, Switzerland; The Moffitt Cancer Center & Research Institute, United States of America

## Abstract

**Purpose:**

Vemurafenib is a potent inhibitor of V600 mutant BRAF with significant impact on progression-free and overall survival in advanced melanoma. Cutaneous side effects are frequent. This single-center observational study investigates clinical and histological features of these class-specific cutaneous adverse reactions.

**Patients and Methods:**

Patients were all treated with Vemurafenib 960 mg b.i.d. within local ethic committees approved clinical trials. All skin reactions were collected and documented prospectively. Cutaneous reactions were classified by reaction pattern as phototoxic and inflammatory, hair and nail changes, keratinocytic proliferations and melanocytic disorders.

**Results:**

Vemurafenib was well tolerated, only in two patients the dose had to be reduced to 720 mg due to arthralgia. 26/28 patients (93%) experienced cutaneous side effects. Observed side effects included UVA dependent photosensitivity (n = 16), maculopapular exanthema (n = 14), pruritus (n = 8), folliculitis (n = 5), burning feet (n = 3), hair thinning (mild alopecia) (n = 8), curly hair (n = 2) and nail changes (n = 2). Keratosis pilaris and acanthopapilloma were common skin reactions (n = 12/n = 13), as well as plantar hyperkeratosis (n = 4), keratoacanthoma (n = 5) and invasive squamous cell carcinoma (n = 4). One patient developed a second primary melanoma after more than 4 months of therapy (BRAF and RAS wild type).

**Conclusion:**

Vemurafenib has a broad and peculiar cutaneous side effect profile involving epidermis and adnexa overlapping with the cutaneous manifestations of genetic diseases characterized by activating germ line mutations of RAS (RASopathy). They must be distinguished from allergic drug reaction. Regular skin examination and management by experienced dermatologists as well as continuous prophylactic photo protection including an UVA optimized sun screen is mandatory.

## Introduction

A variety of unspecific toxicities of cytotoxic agents which emerge in skin, mucosa and adnexa are common. New targeted agents cause class-specific cutaneous side effects.[1]–[6].

An activating BRAF mutation is detected in 40% of melanomas, the most common being BRAF V600 E mutation. Several potent inhibitors of the oncogenic BRAF kinase have been developed and tested in clinical trials. [7], [8] The dose-limiting side effects of these inhibitors include arthralgia, nausea, photosensitivity, fatigue, pruritus and palmar–plantar dysesthesia. In addition, multiple other cutaneous side effects are observed including keratoacanthomas, invasive squamous cell carcinomas and melanomas.[9]–[12].

Vemurafenib (formerly called PLX4032, RG7204, RO5185426) was the first selective BRAF inhibitor to be developed in a clinical setting. This potent inhibitor, orally available, has shown significant impact on both progression-free and overall survival throughout phase I-III clinical trials (BRIM-1, BRIM-2, BRIM-3). [9], [13], [14] Vemurafenib (Zelboraf^©^) has been approved by the FDA, the EMA and in Switzerland. So far, attention regarding the cutaneous side effects of this drug has been mainly devoted to keratinocytic neoplasias such as keratoacanthomas and squamous cell carcinomas, which were seen in 18 to 24% of patients. [9], [13] There is some evidence that the use of a mutation specific BRAF inhibitor leads to a paradoxical activation of the MAPK pathway in cells wild type for BRAF, resulting in cutaneous neoplasias in case of mutations upstream such as RAS. [15] However, far more cutaneous side effects are being observed under treatment and have an important impact on drug tolerance as well as on quality of life.

We investigated the incidence, time point, duration, outcome, clinical presentation and histopathology of this broad spectrum of new class-specific cutaneous side effects induced by Vemurafenib in a cohort of 28 patients undergoing treatment with this drug in clinical trials.

## Patients and Methods

### Patient Selection

A total of 28 patients (15 females, 13 males, age 24–77 years) with metastatic melanoma attending the Dermatology Department of the University Hospital of Zurich and undergoing clinical trials with Vemurafenib during June 2010 until June 2011 formed the study cohort. Written informed consent for inclusion into the trials was obtained from study participants after approval from local ethics committees (Kantonale Ethikkomission Zürich and Swissmedic, see also clinical trials below) as well as the consent to store their information in the hospital database and to use it for research, including publication of photographs (as outlined in the PLoS consent form), (Kantonale Ethikkomission Zürich Biobank/Sammlung von Tumorgewebe, KEK-ZH-Nr. 647).

Data on treatment and all occurring side effects were collected prospectively. Cutaneous reactions were classified by reaction pattern as inflammatory diseases, hair and nail changes, keratinocytic proliferations and melanocytic disorders and proliferations. The appearance of lesions was noted according to treatment duration and was subdivided into three different time intervals of early, intermediate, and late therapy phase (less than 3 weeks, 3 to 6 weeks and more than 6 weeks after therapy start) (Figure 11, 12). A total of 51 Biopsies were collected during the observation period. The biopsies where formalin-fixed, paraffin-embedded and subsequently stained with hematoxylin and eosin or immunochemically stained to detect Ki-67, using MIB-1-Antibody (Dako, Glostrup, Denmark).

### Clinical Trials

Eligible patients with BRAF-mutant melanoma were enrolled in one of the following clinical trials of Vemurafenib (RO5185426, former PLX 4032): Mass balance trial, a Phase I, open-label, excretion balance, pharmacokinetic and metabolism study for a single oral dose of 14C-labeled Vemurafenib in previously treated and untreated patients with metastatic melanoma (Registry: NCT01164891, KEK-ZH-Nr. 2010-0109/5, Swissmedic 2010DR1116); BRIM-3 trial, a randomized, open-label, controlled, multicenter, phase III study in previously untreated patients with unresectable stage IIIC or stage IV melanoma with V600E BRAF mutation receiving Vemurafenib or Dacarbazine (Registry: NCT01006980, KEK-ZH-Nr. 2009-0134/5, Swissmedic 2010DR3078); Roche MO25653 trial, an open-label pilot study of Vemurafenib in previously treated metastatic melanoma patients with brain metastases (Registry: NCT01253564, KEK-ZH-Nr. 2010-0492/0, Swissmedic 2010DR2228) or Roche MO25515 trial, an open-label, multicenter expanded access study of Vemurafenib in patients with metastatic melanoma (Registry: EudraCT Number: 2010-023526-21, KEK-ZH-Nr. 2010-0535/0, Swissmedic 2011DR3030). [13], [16] BRAF testing was performed with the cobas® 4800 BRAF V600 Mutation Test.

All patients were treated with 960 mg b.i.d.; in two patients dose reduction to 720 mg b.i.d was necessary due to side effects, both suffering from arthralgia. Treatment duration lasted from 2 to 12 months. Treatment regimen was stopped when patients experienced progressive disease. No treatment discontinuation was necessary because of side effects.

In 5 of the patients MED (Minimal Erythema Dose) was determined to objectify photosensitivity under Vemurafenib using UV irradiation devices (UVB: emission spectrum from 285 nm to 350 nm (peak at 310–315 nm), UVA 330 nm to 450 nm (peak at 390–410 nm) (Waldmann Lichttechnik, Villingen-Schwenningen, Germany) 10 minutes and 24 hours after irradiation.

## Results

26 patients out of 28 (93%) experienced cutaneous side effects upon drug administration. The emerging adverse reactions were classified by reaction patterns and time of appearance as follows:

### Inflammatory Disorders

#### Photosensitivity reaction

Photosensitivity was observed in 16/28 (57%) patients. In most cases it presented during early phase of therapy. Testing in five patients showed a normal minimal erythema dose (MED) for UVB (range of 0.008 to 0.099 J/cm^2^) in all patients, but a clearly reduced MED for UVA (range 10–49 J/cm^2^) after 10 minutes and 24 hours. In addition, three patients reported burning and pain during UVA exposure. The UV irradiated fields showed a bold erythema including a pronounced edema. [17].

#### Maculopapular exanthema

**Figure 1:**
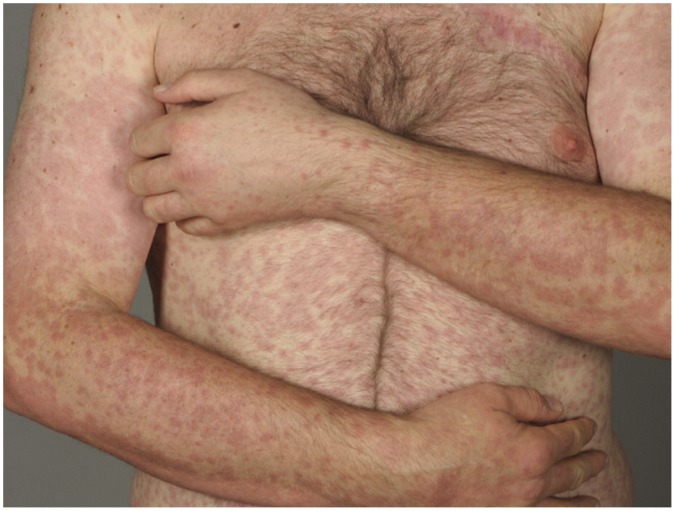
Clinical presentation of the maculopapular rash after 2 weeks of therapy.

**Figure 2:**
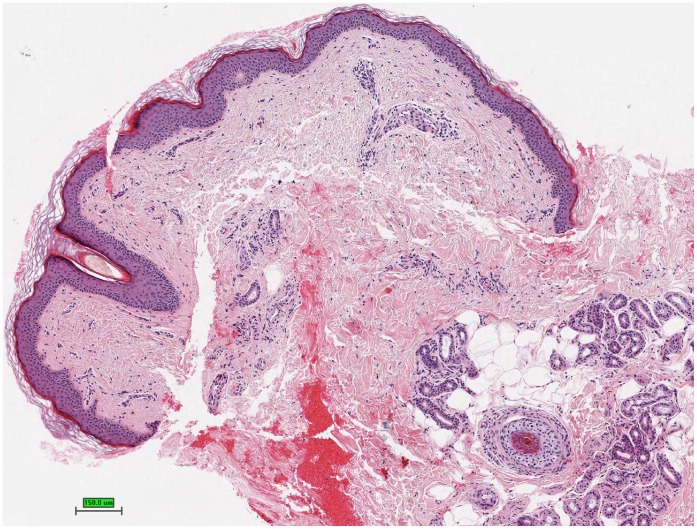
Histology of the maculopapular rash demonstrates a lichenoid lymphohistiocytic infiltrate with interface changes, hematoxylin and eosin stain.

**Figure 3:**
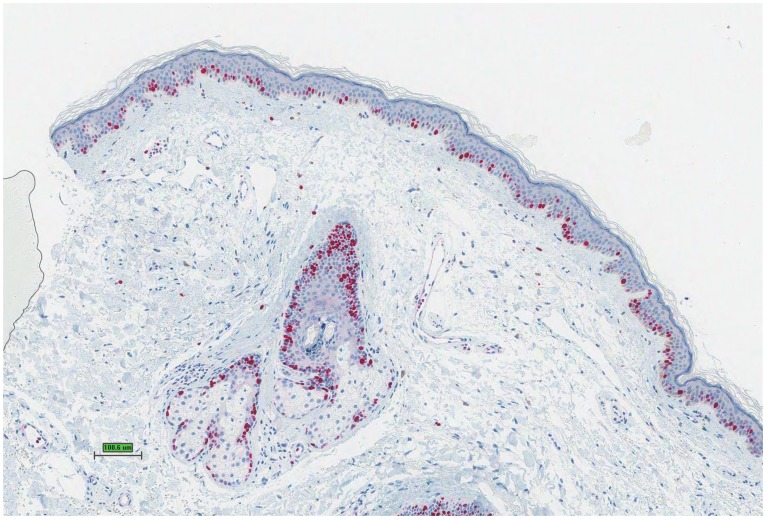
Immunihistological staining for Ki67 in maculopapular rash under treatment with vemurafenib shows increased staining in comparison to normal skin (normal skin not shown).

A maculopapular exanthema was observed 19 times in 14 of 28 patients (50%). The clinical picture showed a disseminated pale erythematous maculopapular rash on the trunk and extremities, less frequent on the face (Figure 1). The majority of cases presented during early and intermediate treatment phase without pruritus and were self-limiting mostly within 4 to 6 weeks. Histologically, these were mostly characterized by vacuolar alteration of the epidermal-dermal junction with a mild perivascular and lichenoid lymphohistiocytic infiltrate with few admixed eosinophils (Figure 2). Mild inflammatory infiltrates around adnexal structures such as hair follicles, sebaceous and eccrine glands were seen in most patients. Interestingly, a lichenoid spongiotic reaction pattern with involvement of adnexal structures was found in one case, whereas in another case a focal granulomatous inflammatory infiltrate and marked syringotropism was noted. Direct immunoflourescence for fibrinogen, C3, IgG, IgA and IgM was negative in all 3 performed biopsies. In comparison to age and site matched healthy skin, immunohistological staining with Ki-67 showed an increasing of the Ki-67-immunoreactivity in the basal layer of the keratinocytes and in the suprabasal and hair follicle keratinocytes. Normal skin shows only a few scattered Ki-67 positive cells in the basal layer (Figure 3). Because of the small sample size no statistical analysis where performed.

#### Follicular rash

Follicular rash or scattered pustules appeared in 5/28 (18%) patients, mostly during intermediate treatment phase.

In two biopsies, a perifollicular and follicular infiltrate of lymphocytes and neutrophils was notable, accompanied by discrete perifollicular fibrosis.

#### Pruritus

Pruritus sine rash was experienced 9 times in a total of 8/28 (29%) patients during the early treatment phase. Pruritus was self-limiting in most cases.

#### Infectious diseases

Infection was an uncommon problem. One patient experienced a disseminated herpes zoster in the late treatment phase under treatment with high dose corticosteroids because of brain metastases. A second patient developed anal herpes simplex in very early treatment stage. Two other patients showed furuncles after more than 6 weeks of treatment.

One patient presented with an acute, diffuse erythema of the leg after 3 weeks on treatment and was diagnosed with cellulitis, responding well to antibiotic treatment. Histologically a perivascular inflammatory infiltrate with numerous neutrophils throughout the whole dermis could be detected. No eosinophils were seen. Overall we do not think that these infectious episodes are specifically related to the Vemurafenib treatment.

### Hair and Nail Changes

Hair thinning and diffuse alopecia occurred in 8/28 (29%) patients. This side effect was observed mostly starting from the third treatment week and continuing thereafter. There was no complete hair loss.

After more than 6 weeks of treatment, 2/28 (7%) patients reported curling of the hair after experiencing hair thinning; the latter was less pronounced later during course of treatment.

Crumbly nails and nail color change was encountered in 2/28 (7%) patients after two weeks and 6 weeks of treatment, respectively.

### Keratinocytic Proliferations

#### Keratosis pilaris

Disseminated small hyperkeratotic follicular papules were noted in 12/28 (43%) patients. This occurred often on the face, proximal upper or lower extremities and was observed more frequently at early treatment time points).

#### Plantar hyperkeratosis

**Figure 4:**
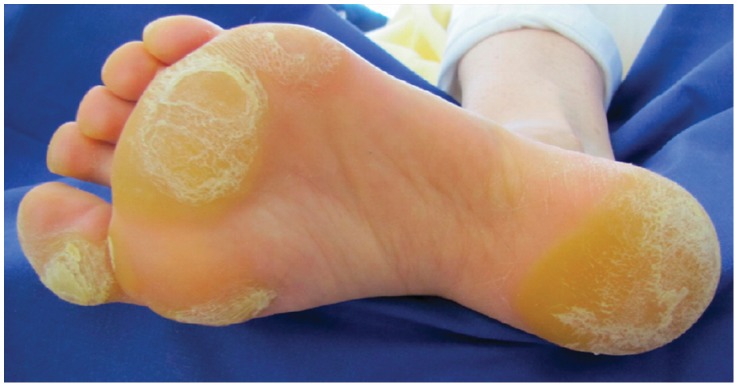
Plantar hyperkeratosis developed after 4 weeks of therapy.

Plantar hyperkeratosis was detected in 4/28 (14%) patients, occurring in areas under physical pressure. All patients but one were more than 6 weeks under treatment with BRAF inhibitor. Additionally, two of these patients described a painful sensation on the hyperkeratotic areas. There was no palmar hyperkeratosis (Figure 4).

One patient reported a painful, burning sensation on the soles with a diffuse erythema, but without hyperkeratosis.

#### Acanthopapilloma

**Figure 5:**
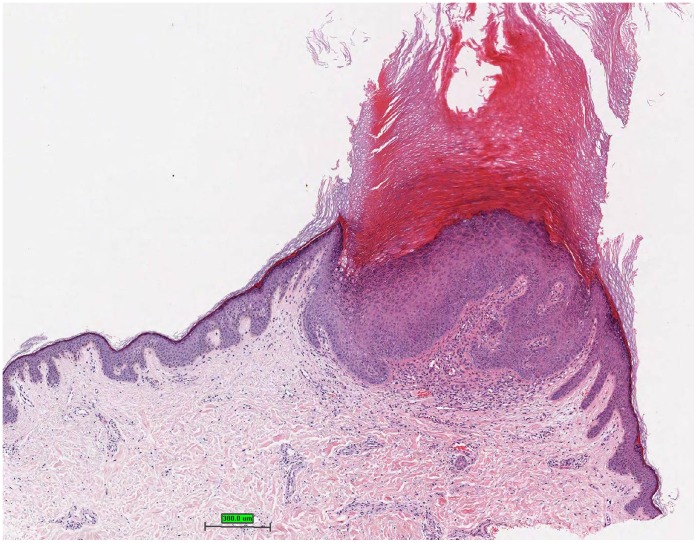
Acanthopapilloma with marked hyperkeratosis and acanthosis, hematoxylin and eosin stain.

Acanthopapillomas (benign acanthotic lesion without signs of malignancy) were a frequently observed dermatologic side effect. 13 out of 28 patients (46%) developed a total of 30 hyperkeratotic papules on the head, neck and trunk, corresponding to acanthopapillomas in the course of therapy. As shown in Figure 12, this side effect occurs at later time points during therapy. Histological evaluation of 30 biopsies of these lesions revealed marked hyperkeratosis and acanthosis with hypergranulosis, koilocytes, mitosis and arborization of the peripheral rete ridges suggesting viral association (Figure 5).

#### Squamous cell carcinoma and keratoacanthoma

Squamous cell carcinomas of keratoacanthoma type were observed in 5/28 (18%) patients in the late phase of treatment.

**Figure 6:**
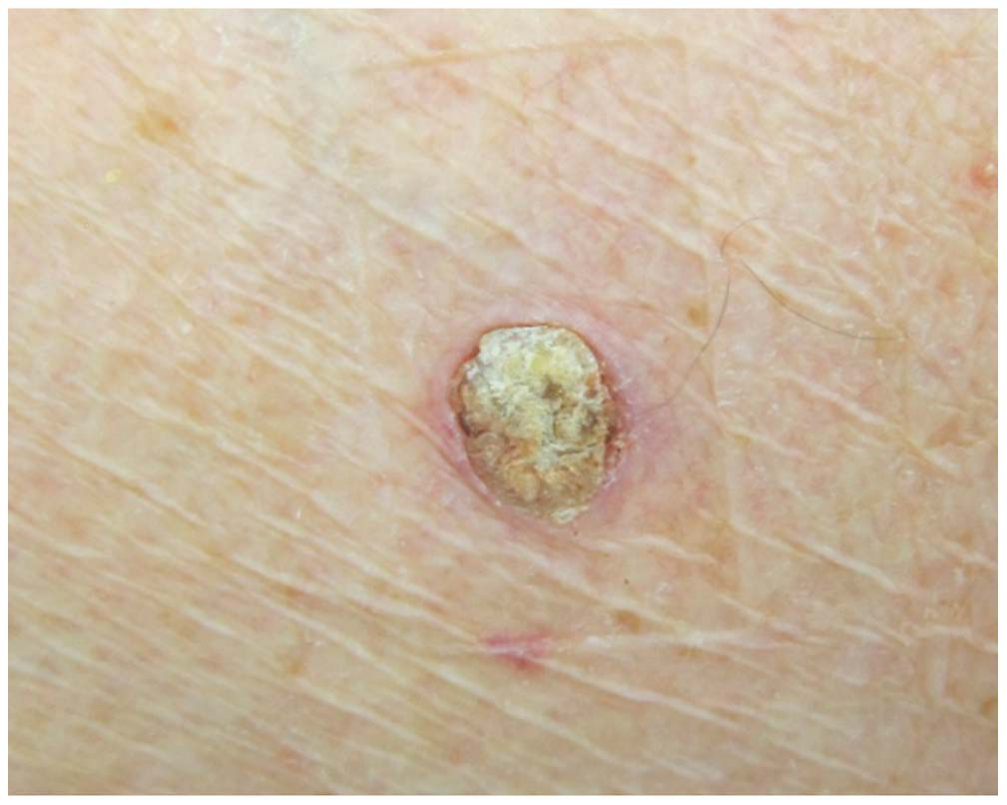
Clinical picture of the kerathoacanthoma, appeared after 5 weeks of treatment.

**Figure 7:**
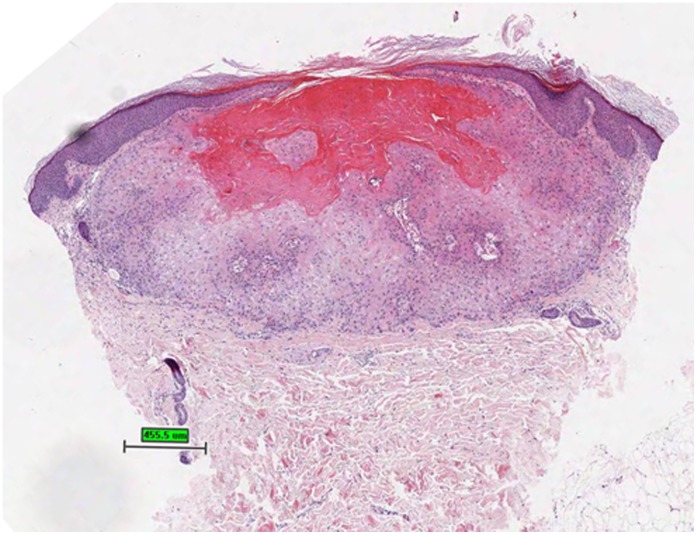
Invagination of keratinizing, squamous epithelium with central keratin-filled crater characterizing a keratoacanthoma, hematoxylin and eosin stain.

Histological features in 5 biopsies were an invagination of keratinizing, well-differentiated squamous epithelium with central keratin-filled crater and symmetrical lipping at the edges of the lesion. 2 cases were diagnosed as keratoacanthoma centrifugum marginatum (Figure 6, 7).

Seven invasive squamous cell carcinomas were observed in 4/28 (14%) patients with photodamaged skin, some with multiple lesions, mostly in the intermediate phase of therapy).

**Figure 8:**
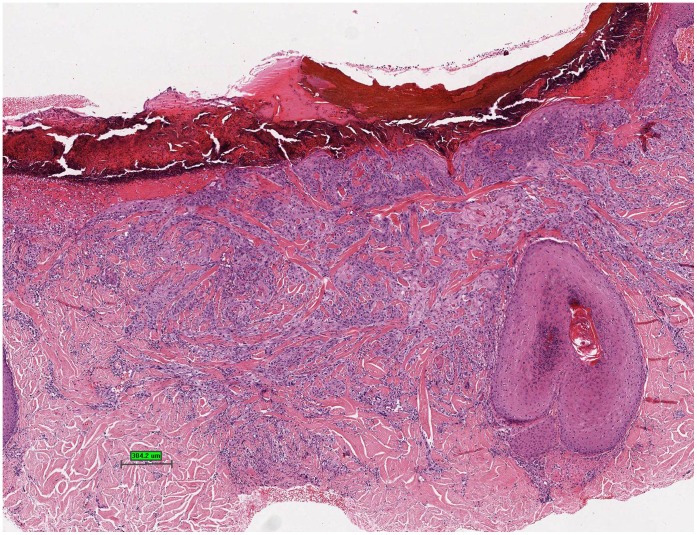
Section of skin with desmoplastic squamous cell carcinoma, hematoxylin and eosin stain.

Histologically, infiltrative growing squamous epithelial cells, mostly well differentiated, could be detected. However, in few cases these tumors were less well differentiated and required several surgical procedures to achieve complete resection. There was one squamous cell carcinoma with an aggressive desmoplastic growth pattern (Figure 8).

Furthermore, the development of Bowen’s Disease, bowenoid squamous cell carcinoma and basal cell carcinoma was observed in single cases as well as a warty dyskerathoma, showing acantholysis and dyskeratosis.

### Melanocytic Disorders and Proliferations

#### Melanoma

**Figure 9:**
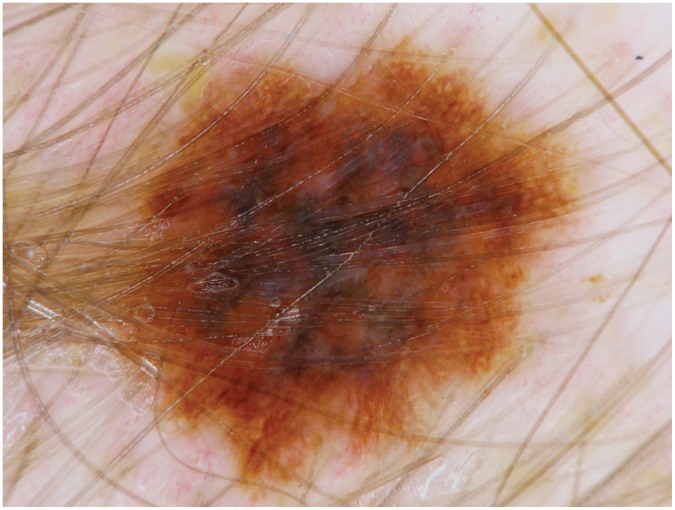
Dermoscopic picture of the melanoma, appeared after more than 4 months of therapy with vemurafenib.

One patient developed a new asymmetrical brownish macule with a sharp border on the capillitium after more than 4 months of therapy. Suspecting a new primary melanoma, the lesion was excised (Figure 9).

**Figure 10:**
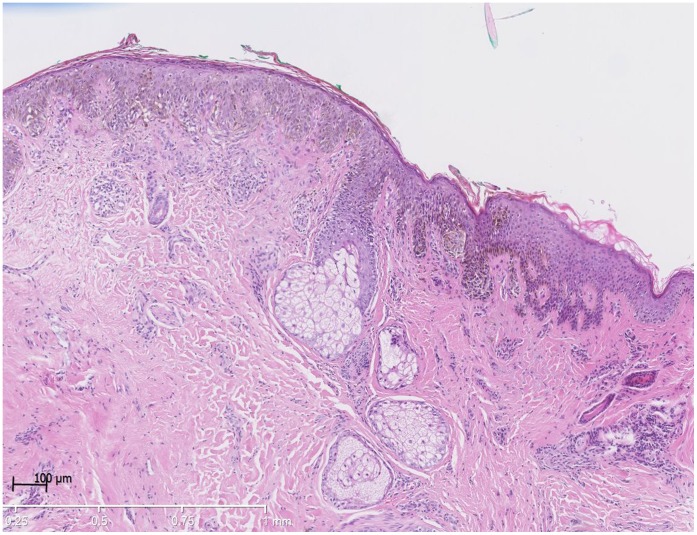
Asymmetric not well circumcised melanocytic proliferation revealing a melanoma with a breslow index of 0.45mm, hematoxylin and eosin stain.

Histology revealed an asymmetric, poorly cicrcumscribed, melanocytic proliferation with atypical melanocytes at the junctional and suprabasal layers as well as in the follicular epithelia. The dermal component of the neoplasm showed no signs of maturation. Peritumoral inflammatory infiltrate was noted. Superficial spreading melanoma, Breslow thickness 0.45 mm, Clark-level III, without ulceration, was diagnosed (Figure 10). There was no evidence of BRAF or NRAS- mutation in this lesion (tested by cobas® 4800 BRAF V600 Mutation Test and polymerase chain reaction sequencing, respectively).

## Discussion

The most common adverse events occurring during Vemurafenib treatment and impacting on the quality of life are skin reactions. So far, no detailed investigation of cutaneous side effects of Vemurafenib accompanied by biopsies has been reported. [10] We analyzed cutaneous side effects under BRAF inhibitors in 28 consecutive patients, focusing on reaction patterns and time of appearance. An attempt to classify these cutaneous adverse events was made in order to facilitate clinical follow-up and diagnosis in a clinical setting.

There was a peculiar sequence of skin reactions with maculopapular exanthema sparing the face in the first four weeks, photosensitivity that occurred in 16 of 28 patients despite the strict recommendation to use sun screens and pruritus in 8 of 28 patients. The photosensitivity is UVA induced and has significant effect on the patient’s quality of life.

**Figure 11:**
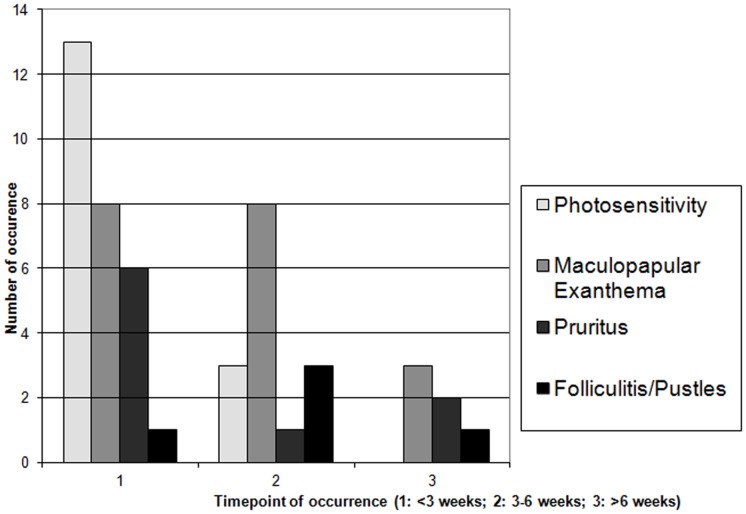
Appearance of keratinocytic proliferations over time during vemurafenib treatment. Timepoint 1∶1–3 weeks; timepoint 2∶3–6 weeks; timepoint 3: more than 6 weeks.

**Figure 12:**
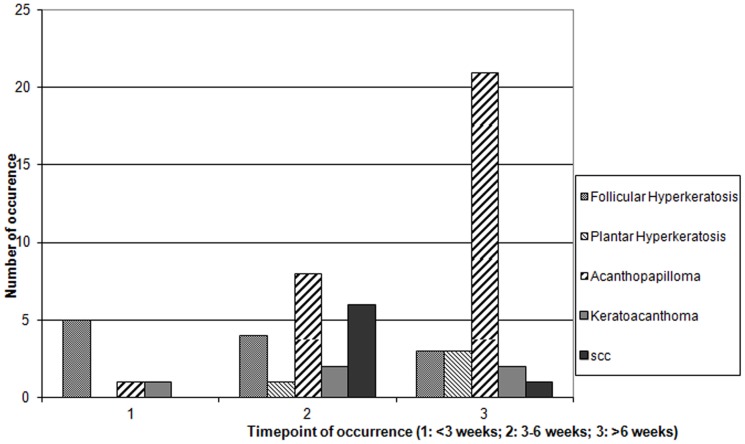
Appearance of inflammatory skin disorders over time during vemurafenib treatment.

Cutaneous reaction 3–6 weeks after treatment initiation (Figure 11, 12) included inflammatory diseases like maculopapular exanthema, folliculitis and others, dystrophic hair- and nail changes and keratinocytic neoplasms. Late side effects (over 6 weeks on treatment) consisted mainly of keratinocytic proliferations, especially acanthopapillomas and keratoacanthomas as well as keratosis pilaris and hair dystrophies.

Alarmingly, second primary melanomas have been found in an unexpected high frequency [18], [19].

The paradox of new malignancies such as keratoacanthoma and squamous cell carcinoma has attracted intensive research. Oberholzer et al. have shown that 21% of squamous cell carcinomas and keratoacanthomas presented activating RAS mutations. [11] In addition, Su et al. confirmed that mutations in RAS, particularly HRAS, are frequent in keratoacanthomas and squamous cell carcinomas in patients treated with Vemurafenib. [20] They have elegantly demonstrated that activated RAS will result in a paradoxical activation of MAPK signaling accelerating tumor growth in BRAF wild type lesions. This suggests that RAS activation is the key event for the progression of keratoacanthomas and squamous cell carcinomas. Homodimer and heterodimer formation involving all members of the BRAF family seems to be involved. [21]–[23].

In case of wild type RAS, an activating signal upstream of RAF such as a mutated EGF-R might drive keratoacanthoma and squamous cell carcinoma proliferation. We have found evidence of a remarkably increased proliferation of keratinocytes shown by immunohistochemistry during the early maculopapular rash, which suggest an increased proliferation rate in the epidermis and follicular structures compared to normal skin.

We argue therefore that other manifestations of the spectrum of skin eruptions depend on RAS activation and therefore might be called RASopathic.

The term RASopathy was introduced to classify a group of syndromes with activating RAS/MAPK germline mutations including cardiofaciocutaneous syndrome (CFC), Costello syndrome (CS), Noonan syndrome and others. [24], [25].

These rare genetic syndromes present multisystem disorders with characteristic coarse facial appearance, intellectual disabilities, tumor predisposition and a spectrum of cutaneous alterations that overlap with Vemurafenib associated skin lesions. [25].

Besides squamous cell carcinomas and keratoacanthomas, many patients present benign keratinocytic neoplasias that are acanthopapillomas or seborrheic keratosis by histology. FGF-R mutations have been found in seborrheic keratosis to be able to activate the pathway. [26] Patients with CS and CFC typically present with acanthopapillomas generally located on the face, especially around the nose.

Keratosis pilaris, plantar pressure dependent hyperkeratosis and dystrophic curly hair with slow growth are common in patients affected by CS or CFC and in Vemurafenib treated patients. The callus like palmo-plantar hyperkeratosis without significant inflammation must be carefully distinguished from the palmoplantar dysethesia syndrome that is a typical and often a dose limiting adverse event during VEGF targeting small molecules such as sunitinib and sorafenib.

Keratosis pilaris, cyst formation and plantar hyperkeratosis might be increased by a dysregulation of the fine tuning of the pathway after physiological stimulation. All the three are classical symptoms of RASopathies.

These cutaneous alterations in addition to squamous cell carcinoma and keratoacanthoma are also observed during sorafenib therapy. [3] This paper suggests an induction of keratinocytic hyperproliferation by sorafenib without signs of apoptosis resulting in increased epidermal thickness in normal skin. This probably contributes to the clinical presentations of keratosis pilaris and plantar hyperkeratosis. It appears as the opposite of the reaction pattern induced by MEK inhibitors. MEK inhibition results in a suppression of the pathway in keratinocytes resulting in a stress reaction with up-regulation of p53 and a release of cytokines attracting inflammatory cells with the clinical presentation of the typical maculopapular and pustular rash observed during the use of MEK inhibitors. [1].

The dystrophic hair growth with curly thin hairs often associated with a change of the hair color is often referred to as alopecia. However, it is definitely different to the hair loss seen during chemotherapy. It occurs slowly and complete baldness was not observed in our patients.

As the number of Vemurafenib-treated patients will increase in the near future, it is important to understand the cutaneous side effects of this drug. It is essential to distinguish them from allergic drug reactions. Further classification will facilitate the development of follow-up schedules and clinical management. We propose to conduct detailed dermatological examinations of the skin every 4 weeks during Vemurafenib therapy. Patients should be well informed of the expected cutaneous side effects, especially about the UVA photosensitivity. The use of UVA optimized sunscreens and UV blocking clothing should be strongly recommended, since testing and experience show a prophylactic effect. [17] In the future, topical application of the vitamin D derivate calcipotriol and/or retinoids appears promising to normalize the epidermal hyperproliferation. [27], [28] Further there are observations of decreased appearing of inflammatory and neoplastic skin lesions when BRAF inhibitors like vemurafenib are combined with MEK Inhibitors. [29] Placebo controlled trials will help to investigate the benefit of these co-medications.
