# Correction: RASopathic Skin Eruptions during Vemurafenib Therapy

**DOI:** 10.1371/annotation/d884b668-04f7-4ae6-96a3-a199df5b43be

**Published:** 2013-10-09

**Authors:** Jeannine D. Rinderknecht, Simone M. Goldinger, Sima Rozati, Jivko Kamarashev, Katrin Kerl, Lars E. French, Reinhard Dummer, Benedetta Belloni

The first two authors, Jeannine D. Rinderknecht and Simone M. Goldinger, should be noted as contributing equally to this work. 

